# Survey design and analysis considerations when utilizing misclassified sampling strata

**DOI:** 10.1186/s12874-021-01332-8

**Published:** 2021-07-11

**Authors:** Aya A. Mitani, Nathaniel D. Mercaldo, Sebastien Haneuse, Jonathan S. Schildcrout

**Affiliations:** 1 Division of Biostatistics, University of Toronto Dalla Lana School of Public Health, Toronto, Canada; 2 Department of Neurology, Massachusetts General Hospital, Boston, USA; 3 Department of Biostatistics, Harvard T.H. Chan School of Public Health, Boston, USA; 4 Department of Biostatistics, Vanderbilt University Medical Center, Nashville, USA

**Keywords:** Complex survey, Disproportionate stratified sampling, Stratum misclassification, Design-based analysis, Model-based analysis

## Abstract

**Background:**

A large multi-center survey was conducted to understand patients’ perspectives on biobank study participation with particular focus on racial and ethnic minorities. In order to enrich the study sample with racial and ethnic minorities, disproportionate stratified sampling was implemented with strata defined by electronic health records (EHR) that are known to be inaccurate. We investigate the effect of sampling strata misclassification in complex survey design.

**Methods:**

Under non-differential and differential misclassification in the sampling strata, we compare the validity and precision of three simple and common analysis approaches for settings in which the primary exposure is used to define the sampling strata. We also compare the precision gains/losses observed from using a disproportionate stratified sampling scheme compared to using a simple random sample under varying degrees of strata misclassification.

**Results:**

Disproportionate stratified sampling can result in more efficient parameter estimates of the rare subgroups (race/ethnic minorities) in the sampling strata compared to simple random sampling. When sampling strata misclassification is non-differential with respect to the outcome, a design-agnostic analysis was preferred over model-based and design-based analyses. All methods yielded unbiased parameter estimates but standard error estimates were lowest from the design-agnostic analysis. However, when misclassification is differential, only the design-based method produced valid parameter estimates of the variables included in the sampling strata.

**Conclusions:**

In complex survey design, when the interest is in making inference on rare subgroups, we recommend implementing disproportionate stratified sampling over simple random sampling even if the sampling strata are misclassified. If the misclassification is non-differential, we recommend a design-agnostic analysis. However, if the misclassification is differential, we recommend using design-based analyses.

**Supplementary Information:**

The online version contains supplementary material available at (10.1186/s12874-021-01332-8).

## Background

Health research increasingly relies on data from large biobanks that contain biological samples and genomic data that are linked to clinical information through electronic health records (EHR). Historically, however, patients involved in health research including clinical trials and genetic studies have been comprised mostly of individuals with northern European ancestry [1–4]. Creating a more diverse cohort of patients in health research has been a widely recognized goal in the recent years [5]. In order to better understand the concerns about, and barriers to participating in biobank-derived research among the underrepresented groups, the Consent, Education, Regulation and Consultation (CERC) working group of the electronic Medical Records and Genomics (eMERGE) network conducted a large multi-site survey. The target population of the survey was patients who had an inpatient or outpatient visit at one of the eleven eMERGE network clinical centers between October 1, 2013 and September 30, 2014, had a geocodable residential address, and had age and gender available in the EHR. The CERC researchers used disproportionate stratified sampling (DSS) to enrich the sample with racial and ethnic minorities, younger adults, and patients of low socio-economic status. Specifically, they used age, gender, race, ethnicity, education, and rural living, obtained from EHR, supplemented by US Census data, to identify the sample. Further details on survey design and results are provided in previous publications [6, 7].

The CERC survey included a number of questions that aimed to understand patients’ willingness to participate in biobank-derived research, their safety and privacy concerns and overall trust in the healthcare system. Respondents also provided self-reported demographic variables including race and ethnicity [7]. However, a fraction of the patients’ self-reported race and ethnicity differed from the EHR-derived race and ethnicity that were used to create the sampling strata.

Inaccurate measurements of exposure or outcome variables are commonly referred to as misclassification for categorical variables and mismeasurement for continuous variables. The impact of and possible solutions for misclassification and mismeasurement in standard regression settings have been studied extensively [8–10]. In the CERC substudy, however, misclassification occurred in the variables that defined the sampling strata. Furthermore, the misclassification in the sampling strata was differential (i.e., presence or absence of misclassification is associated with the outcome variable itself). To our knowledge the impact of misclassified sampling strata (differential or non-differential) in complex survey designs is not well studied.

In this paper, we investigate sampling strata misclassification when implementing a complex survey design. In particular, we are interested in characterizing the impact of varying degrees of non-differential and differential sampling strata misclassification on the operating characteristics of commonly used estimation procedures including: model-based, design-based and the seemingly naïve, design-agnostic procedures. We also draw comparisons with random sampling procedures.

### Motivating study

The eMERGE Network CERC survey was administered to 11 US (adult) clinical centers to understand patients’ views regarding consent and data sharing models for genomics research, especially among racial and ethnic minorities, as well as younger adults, individuals of low socio-economic status, rural residence and low education attainment level. Disproportionate stratified sampling was used to enrich the final sample with subjects from small-size strata by applying unequal sampling probabilities to each sampling stratum. In the original CERC survey, the cross-classification of six variables: age, gender, race, ethnicity, educational attainment and rural living, was used to define the sampling strata, with a maximum entropy sampling algorithm to define the sampling probabilities [7].

For the purpose of examining the impact of sampling strata misclassification in an exposure-enriched design, we restrict our sample to patients from Vanderbilt University Medical Center (VUMC). The primary analysis in this paper seeks to characterize the association between patient trust in the healthcare system and a number of patient demographics including race/ethnicity, age, gender, rural residence, education level and income. Note that in contrast to the other variables, income was not used to create the sampling strata. Trust in the healthcare system was defined as 1 if the respondent answered either “strongly agree" or “agree” to the statement “I trust my healthcare system”, and 0 otherwise.

We will focus on the race/ethnicity variable to describe the misclassification that occurred between the EHR system and the self-reported survey, and assume that the self-reported race/ethnicity is the gold-standard. In the EHR system, 86.3% of the 329,672 patients were recorded as Non-Hispanic White (hereon referred to as White), 9.5% as Non-Hispanic Black (hereon referred to as Black), 1.2% as Asian, 0.9% as Other, and 2.1% as Hispanic. In Table 1, we present the misclassification matrix among all survey respondents and the misclassification matrix stratified by the outcome, trust in the healthcare system. Among the respondents recorded as “White” and “Black” in the EHR system, most (94.4% and 93.7%) also reported themselves as “White” and “Black” in the survey response. On the other hand, among the respondents recorded in the EHR system as “Other” and “Hispanic”, only 27.6% and 53.1% self-reported as “Other” and “Hispanic” respectively.

**Table 1:** Misclassification matrix among Vanderbilt University Medical Center respondents overall and by trust in the healthcare system. Cell values indicate number of respondents and those in parentheses denote row percentages by strata

	Self-reported race/ethnicity				
EHR-based race/ethnicity	White	Black	Asian	Other	Hispanic
**Overall**					
White	134 (94.4)	1 (0.7)	0 (0.0)	6 (4.2)	1 (0.7)
Black	0 (0.0)	74 (93.7)	0 (0.0)	4 (5.1)	1 (1.3)
Asian	1 (1.2)	1 (1.2)	62 (76.5)	14 (17.3)	3 (3.7)
Other	59 (48.0)	5 (4.1)	16 (13.0)	34 (27.6)	9 (7.3)
Hispanic	43 (24.0)	29 (16.2)	3 (1.7)	9 (5.0)	95 (53.1)
**Stratified**					
Trust = 0					
White	35 (97.2)	0 (0.0)	0 (0.0)	0 (0.0)	1 (2.8)
Black	0 (0.0)	20 (87.0)	0 (0.0)	2 (8.7)	1 (4.3)
Asian	0 (0.0)	0 (0.0)	25 (78.1)	6 (18.8)	1 (3.1)
Other	28 (56.0)	1 (2.0)	3 (6.0)	14 (28.0)	4 (8.0)
Hispanic	11 (18.0)	9 (14.8)	1 (1.6)	3 (4.9)	37 (60.7)
Trust = 1					
White	99 (93.4)	1 (0.9)	0 (0.0)	6 (5.7)	0 (0.0)
Black	0 (0.0)	54 (96.4)	0 (0.0)	2 (3.6)	0 (0.0)
Asian	1 (2.0)	1 (2.0)	37 (75.5)	8 (16.3)	2 (4.1)
Other	31 (42.5)	4 (5.5)	13 (17.8)	20 (27.4)	5 (6.8)
Hispanic	32 (27.1)	20 (16.9)	2 (1.7)	6 (5.1)	58 (49.2)

Misclassification rates varied when stratified by trust in the healthcare system, suggesting that misclassification was differential, i.e. the degree of strata misclassification varied according to the outcome of interest (Table 1). For example, among those who were recorded as Black in the EHR system, 87.0% (Trust = 0) and 96.4% (Trust = 1) self-reported as Black, and among those who were recorded as Hispanic in the EHR system, 60.7% (Trust = 0) and 49.2% (Trust = 1) self-reported as Hispanic.

## Methods

The primary goal of a survey is to accurately estimate quantities such as totals or means, or to describe the relationship among variables through fitting a statistical model using a subsample from the finite target population. Suppose interest lies in understanding the relationship between a binary outcome *Y* and a categorical exposure *X* with *H* categories. We denote *X*_*h*_ as the indicator variable for the *h*th category such that *X*_*h*_=1 if *X*=*h* and *X*_*h*_=0 otherwise, for *h*=1,...,*H*. Due to resource constraints, *Y* and *X* can only be ascertained from a subsample of individuals. However, assume the distribution of *X* in the target population is unbalanced, e.g., if *H*=3 with Pr(*X*_1_=1,*X*_2_=0,*X*_3_=0)=0.8 and Pr(*X*_1_=0,*X*_2_=1,*X*_3_=0)= Pr(*X*_1_=0,*X*_2_=0,*X*_3_=1)=0.1, then taking a random sample of individuals will result in few individuals in the low prevalence groups (*X*_2_ and *X*_3_).

In order to have enough individuals from each of the *h*=1,...,*H* categories of X in the subsample to make meaningful inference, survey sampling methods suggest to oversample the individuals in the low prevalence categories. Now suppose *X* is misclassified and denote the misclassified version of *X* as *X*^∗^ which means that sampling will be based on *X*^∗^ instead of *X*. Here, *X*^∗^ also has *H* categories and we denote $X_{h}^{\ast }$ as the indicator variable for the *h*th category for *h*=1,...,*H*. However, note that, due to misclassification, Pr(*X*_*h*_=1) does not necessarily equal $\Pr (X_{h}^{\ast }=1)$. For simplicity, we let the sampling design based on *X*^∗^ consist of *h*=1,...,*H* strata corresponding to the *h*=1,...,*H* categories of *X*^∗^. Finally, we assume that we can ascertain the true exposure *X* with the outcome *Y* when participants respond to the survey. Thus, we will observe all three variables (*Y,X*,*X*^∗^) for all individuals in the subsample. In the CERC example, *Y* corresponds to trust in the healthcare system, *X* corresponds to self-reported race/ethnicity, and *X*^∗^ corresponds to EHR-based race/ethnicity.

In the next two sections, using directed acyclic graphs (DAGs), we describe the implications of applying three common methods for analyzing data sampled from a DSS design in the presence of non-differential and differential misclassification of the sampling variable. In addition to (*Y,X*,*X*^∗^), we also have *S* to represent the binary sampling indicator [11] which is set to 1 if sampled and 0 if not.

### Non-differential misclassification of *X*^∗^

**Fig. 1:**
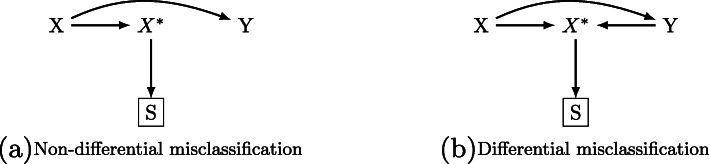
Directed acyclic graphs (DAGs) representing disproportionate stratified sampling in the presence of non-differential and differential misclassification

Figure 1a depicts exposure enriched sampling based on *X*^∗^ in the presence of non-differential misclassification. Under non-differential misclassification, the misclassification of *X* is independent of the true outcome (*X*^∗^⊥ ⊥*Y*|*X*) which means that *X*^∗^ is affected only by *X*, and there is no direct or indirect relationship between *X*^∗^ and *Y*. The association of interest is *X*→*Y*.

For a binary *Y*, the naïve method of analysis is to ignore the complex survey design and fit a standard logistic regression model, using *X*_1_ as the reference category, such as:

1 $$ \text{logit}\left\{\Pr(Y=1|X)\right\}=\beta_{0} + \sum_{h=2}^{H}\beta_{1h}X_{h}.  $$

In this paper, we will call this the design-agnostic analysis. Under the non-differential misclassification scenario depicted in Fig. 1a, this seemingly naïve method that doesn’t account for the survey design still produces consistent estimates (that is, estimates for which bias decreases to zero as the sample size increases) for *β*_0_ and *β*_12_,...,*β*_1*H*_ because Pr(*Y*=1|*X,S*=1)= Pr(*Y*=1|*X*).

To show this, note that

$$\begin{aligned} \Pr(S=1|Y,X)&=\sum_{X^{\ast}}\Pr(S=1|X^{\ast},X,Y)\Pr(X^{\ast}|X,Y)\\ &=\sum_{X^{\ast}}\Pr(S=1|X^{\ast})\Pr(X^{\ast}|X)\\ &=\Pr(S=1|X). \end{aligned} $$

Therefore,

$$\begin{aligned} \Pr(Y=1|X, S=1)&=\frac{\Pr(S=1|Y,X)\Pr(Y=1|X)}{\Pr(S=1|X)}\\ &=\Pr(Y=1|X). \end{aligned} $$

Another option is to account for the survey design by including *X*^∗^ as a covariate in Eq. 1:

2 $${} \text{logit}\left\{\Pr(Y=1|X, X^{\ast})\right\}=\beta_{0} + \sum_{h=2}^{H}\beta_{1h}X_{h} + \sum_{h=2}^{H}\beta_{2h}X^{\ast}_{h}.  $$

This is the classical model-based approach where the sampling variable is included as a covariate in the regression model to account for the survey design. Because *X*^∗^⊥ ⊥*Y*|*X*, the parameter estimates for *β*_22_,...,*β*_2*H*_ will be close to zero. The estimates for *β*_12_,...,*β*_1*H*_ will be consistent but we expect standard errors will be larger compared to those from the design-agnostic model due to the likely high degree of correlation between *X* and *X*^∗^. Furthermore, under the non-differential misclassification scenario, *β*_12_,...,*β*_1*H*_ in Eqs. 1 and 2 are equivalent. This is because *X*^∗^ is unrelated to *Y*, and therefore the association between *Y* and *X* is collapsible across *X*^∗^.

A third option is to account for the survey design by incorporating sampling probability weights based on *X*^∗^. To do this, we compute the selection probability for each individual in the subsample. Let $N^{\ast }_{h}$ denote the number of individuals in stratum *h* and suppose $N^{\ast }_{h}$ individuals are sampled from each of the *h*=1,...,*H* strata. Then, the selection probability for an individual *i* belonging in stratum *h* is $\frac {n^{\ast }_{h}}{N^{\ast }_{h}}$. Let $w_{i} = \left (\frac {n^{\ast }_{h}}{N^{\ast }_{h}}\right)^{-1}$ be the sampling probability weight for individual *i* belonging in stratum *h*. A regression model that incorporates the sampling probability weights is referred to as model-assisted design-based analysis, which we define as design-based analysis. The parameters of the design-based model are those that maximize the following weighted joint log-likelihood [12]:

3 $${} \sum_{i}w_{i}~l_{i}(\boldsymbol{\beta}|\boldsymbol{x}_{i}) = \sum_{i}w_{i}\left\{y_{i}\text{log}(p_{i})+(1-y_{i})\text{log}(1-p_{i})\right\}  $$

where $p_{i} = p_{i}(\boldsymbol {x}_{i};\boldsymbol {\beta })= \frac {\exp \left (\beta _{0}+\sum _{h}\beta _{1h}x_{hi}\right)}{1+\exp \left (\beta _{0}+\sum _{h}\beta _{1h}x_{hi}\right)}$. Under non-differential misclassification, the design-based method will produce valid parameter estimates. However, we expect the standard errors will be larger compared to estimates from a model without weights (i.e. design-agnostic method in Eq. 1) [13].

### Differential misclassification of *X*^∗^

Figure 1b depicts the same exposure enrichment sampling but in the presence of differential misclassification. Under this scenario, because *X*^∗^ is dependent on *Y* in addition to *X*, another arrow exists between *Y* and *X*^∗^. In the literature, the type of bias introduced by *Y*→*X*^∗^ is called information bias or measurement bias [14]. In a typical observational study where only *X*^∗^ is available as a surrogate of *X*, information bias is problematic as there is no analytical remedy unless one seeks to conduct a validation study [15]. In our study, however, we eventually ascertain *X* through survey response so we observe all *Y*, *X* and *X*^∗^. In such instances, *X*^∗^ becomes a collider which is a variable influenced by two other variables [16].

Under differential misclassification of *X*^∗^, both the design-agnostic and model-based methods explained above produce biased parameter estimates. The design-agnostic method is no longer appropriate because Pr(*Y*=1|*X,S*=1)≠ Pr(*Y*=1|*X*) and the model in Eq. 1 fails to account for the survey design. Controlling for the sampling variable *X*^∗^ as done in model-based analysis in Eq. 2 is also inappropriate because stratification by a collider will distort the true association of *X*→*Y* and result in biased parameter estimates of *X* [16]. Hence, only the design-based method is able to produce valid estimates of *X*. The design-based method appropriately accounts for the survey design by incorporating sampling probability weights and modeling the direct association of *X*→*Y*. The construction of sampling probability weights, *w*_*i*_, under differential misclassification of *X*^∗^ is equivalent to when misclassification is non-differential.

In the next section, we show how the three methods described above perform under non-differential and differential misclassification settings through a simulation study.

## Simulation study

### Design

We conducted a simulation study to investigate the effect of sampling variable misclassification on the validity and precision of parameter estimates from design-agnostic, model-based, and design-based analyses. In addition to evaluating the parameter estimates of a sampling variable used to construct the sampling strata, race/ethnicity, we also assess the validity and efficiency of a non-sampling variable, low-income, defined here as income < $30,000. For each simulation iteration, we generated a population data of size *N*=100,000 from the model:

4 $$\begin{array}{*{20}l} \Pr(\text{Trust}_{i} &= 1|\text{race/ethnicity}_{i}, \text{low-income}_{i})\\ &= \text{logit}^{-1}[\beta_{0} + \beta_{B}I(\text{Black}_{i}) + \beta_{A}I(\text{Asian}_{i}) \\ &+ \beta_{O}I(\text{Other}_{i}) + \beta_{H}I(\text{Hispanic}_{i}) \\ &+ \beta_{L}I(\text{low-income}_{i})], \end{array} $$

where *I* represents the indicator function and (*β*_0_,*β*_*B*_,*β*_*A*_,*β*_*O*_,*β*_*H*_,*β*_*L*_)=(−0.75,−0.25,−0.50,1.25,−1.50,1.00). We used the following proportion of the true race/ethnicity (*X*) in the population: (White, Black, Asian, Other, Hispanic) =(0.82,0.10,0.01,0.05,0.02) which was based on the CERC study’s survey-weighted population proportions estimated from the sample. We generated low-income from the model:

5 $$\begin{array}{*{20}l}{} \Pr(\text{Low-income}_{i}&= 1|\text{race/ethnicity}_{i})\\ &= \text{logit}^{-1}\Big[-2.00 + 1.25I(\text{Black}_{i}) \\ & + 0.25I(\text{Asian}_{i}) \\ & + 1.75I(\text{Other}_{i}) + 0.50I(\text{Hispanic}_{i})\Big]. \end{array} $$

We then created two types of misclassified race (*X*^∗^) variable:

1. Non-differential misclassification based on the overall misclassification matrix in Table 1
2. Differential misclassification based on the stratified misclassification matrices in Table 1

We drew a sample of size *n*=2500 from the population using simple random sampling (SRS) and DSS. For DSS, we sampled 500 individuals from each of the five potentially misclassified race categories.

For the sample obtained from DSS, we conducted three different types of analyses described in the Methods section. For the design-based method, we incorporated sampling weights constructed from the misclassified race variable in the logistic regression models described in Eq. 4. For the model-based method, we included the misclassified race as an additional covariate in the logistic regression models. Finally, for the design-agnostic method, we ignored the design information and did not incorporate any sampling weights or adjust for the sampling variable in the logistic regression models. All analyses were performed in R using the base [17] and survey packages [18].

For each type of misclassification mechanism, sampling design, and analytical method, we performed a simulation with a total of 10,000 iterations. For each iteration, we collected the parameter estimates and standard errors and present the mean parameter estimates, empirical standard errors (SEs; i.e. standard deviations of each parameter estimates), and 95% coverage probabilities.

## Results

### Simulation study

Table 2 shows the mean parameter estimates, empirical SEs and 95% coverage probabilities for the full cohort, sample obtained by SRS and by DSS under non-differential and differential misclassification. Under the DSS design, simulation results from three different methods are displayed: design-agnostic (based on Eq. 1), model-based (based on Eq. 2), and design-based (based on Eq. 3) analyses.

**Table 2:** Simulation results: Means, empirical standard errors and 95% coverage probabilities of parameter estimates from 10,000 simulations under observed misclassification rates of race/ethnicity by sampling design and method

	Full cohort		SRS		Disproportionate stratified sampling					
					Design-agnostic		Model-based		Design-based	
**Non-differential misclassification**										
Intercept	-0.75 (0.01)	94.7	-0.75 (0.05)	95.0	-0.75 (0.06)	95.1	-0.75 (0.10)	94.5	-0.75 (0.07)	94.9
Black	-0.25 (0.02)	95.0	-0.26 (0.15)	94.9	-0.25 (0.11)	94.8	-0.26 (0.25)	95.4	-0.25 (0.12)	94.9
Asian	-0.50 (0.07)	95.0	-0.59 (0.82)	96.7	-0.51 (0.21)	95.2	-0.51 (0.26)	95.2	-0.52 (0.27)	93.0
Other	1.25 (0.03)	95.1	1.26 (0.21)	95.2	1.25 (0.14)	95.5	1.26 (0.18)	95.6	1.26 (0.21)	95.1
Hispanic	-1.50 (0.07)	95.0	-1.63 (0.87)	96.8	-1.55 (0.41)	95.9	-1.55 (0.42)	96.0	-1.56 (0.47)	95.2
Low income	1.00 (0.02)	94.5	1.00 (0.12)	95.4	1.00 (0.11)	95.1	1.01 (0.11)	95.1	1.00 (0.14)	95.0
**Differential misclassification**										
Intercept	-0.75 (0.01)	94.7	-0.75 (0.05)	95.1	-0.57 (0.06)	13.0	-0.74 (0.10)	95.0	-0.75 (0.07)	95.2
Black	-0.25 (0.02)	95.0	-0.25 (0.15)	95.1	-0.42 (0.11)	67.6	-0.42 (0.24)	90.1	-0.26 (0.12)	94.8
Asian	-0.50 (0.07)	95.0	-0.58 (0.79)	96.5	-0.81 (0.20)	67.9	-2.16 (0.27)	0.0	-0.52 (0.28)	91.7
Other	1.25 (0.03)	95.1	1.26 (0.21)	95.0	0.96 (0.13)	42.4	0.02 (0.19)	0.0	1.25 (0.20)	94.5
Hispanic	-1.50 (0.07)	95.0	-1.62 (0.87)	97.0	-1.63 (0.36)	96.3	-2.14 (0.38)	62.1	-1.55 (0.42)	95.2
Low income	1.00 (0.02)	94.5	1.00 (0.12)	94.7	1.00 (0.11)	95.2	1.01 (0.11)	94.9	1.00 (0.15)	94.8

#### Non-differential misclassification

Under non-differential misclassification (top half of Table 2), logistic regression estimates from SRS were unbiased for the intercept and the non-sampling variable (low-income). Black and Other parameter estimates also had very little bias while Asian and Hispanic (smallest subgroups) estimates had some bias. The biases occurred because in some simulations, the sample from SRS had extremely few Asian or Hispanic respondents. Due to the very low prevalence of Asian and Hispanic patients in the population, the SRS scheme can fail to include enough respondents from rare subgroups to make meaningful inference. As expected, empirical SEs of all SRS association estimates were larger than association estimates from DSS designs, except for the intercept which represents the most prevalent subgroup (White) and Black. Within SRS, the empirical SEs of the rare subgroup estimates, Asian and Hispanic were especially large (0.82 and 0.87 respectively) compared to the other estimates, also reflecting the high 95% coverage probabilities (96.7 and 96.8% respectively).

All three methods under the DSS scheme yielded estimates with very low bias. We observed more notable differences in the empirical SEs across the methods. Also as expected, the design-agnostic analysis without weights or adjustment, produced the most precise estimates with lowest SEs across all variables. Whereas the SEs of the sampling variable (race/ethnicity) from the model-based analysis were larger compared to those from the design-agnostic analysis, the SEs of the non-sampling variable (low-income) were comparable. In general, we observed larger SEs under the design-based analysis compared to the design-agnostic, particularly for the rare subgroups (Asian, Other, Hispanics). This is not surprising as the inverse sampling probability weights associated with the rare subgroups are larger than those associated with the more prevalent subgroups. For example, the SEs for the Hispanic parameter estimates from the design-agnostic, model-based and design-based methods were 0.41, 0.42, and 0.47 respectively. The SE of the non-sampling variable was also slightly larger from the design-based method (0.14) compared to the design-agnostic (0.11) or the model-based (0.11) method.

### Differential misclassification

Under differential misclassification, simulation results from SRS were similar to those under non-differential misclassification. This is not surprising because the SRS probability is independent of *Y* or *X*^∗^. However, the design-agnostic and the model-based methods produced substantial biases in the sampling variable (race/ethnicity) estimates with lower coverage probabilities, especially for the rare subgroups (Asian, Other, Hispanic). On the other hand, the design-based method produced unbiased sampling variable estimates with coverage probabilities close to 95%. For example, the estimates for the Asian parameter (true value of -0.50) were -0.81 and -2.16 for the design-agnostic and model-based methods respectively, while it was -0.52 for the design-based method. And the 95% coverage probabilities were 67.9% and 0.0% for the design-agnostic and model-based methods respectively, while it was 91.7% for the design-based method. The parameter estimates for low-income remained unbiased with coverage probabilities close to 95% for all three DSS methods.

### Relative uncertainty of disproportionate stratified sampling compared to simple random sampling under non-differential misclassification

We evaluated the empirical SEs of the logistic regression parameter estimates for the design-agnostic, model-based and design-based methods under various degrees of non-differential misclassification. We varied the degree of misclassification from 0, where all patients’ race are correctly classified (no misclassification), to 0.5, where half of the patients’ race are misclassified. For each degree of misclassification, we repeated the simulation using the same design explained earlier. We then computed the empirical SE of each parameter estimate from each of the three methods. Note that we focus on evaluating the uncertainty of the parameter estimates under non-differential misclassification because each method yielded unbiased estimates and as part of assessing overall model performances, we wanted to additionally examine precision. We did not further evaluate uncertainty under differential misclassification because the design-based method produced considerably less biased estimates relative to the design-agnostic or model-based method under various degrees of misclassification.

**Fig. 2:**
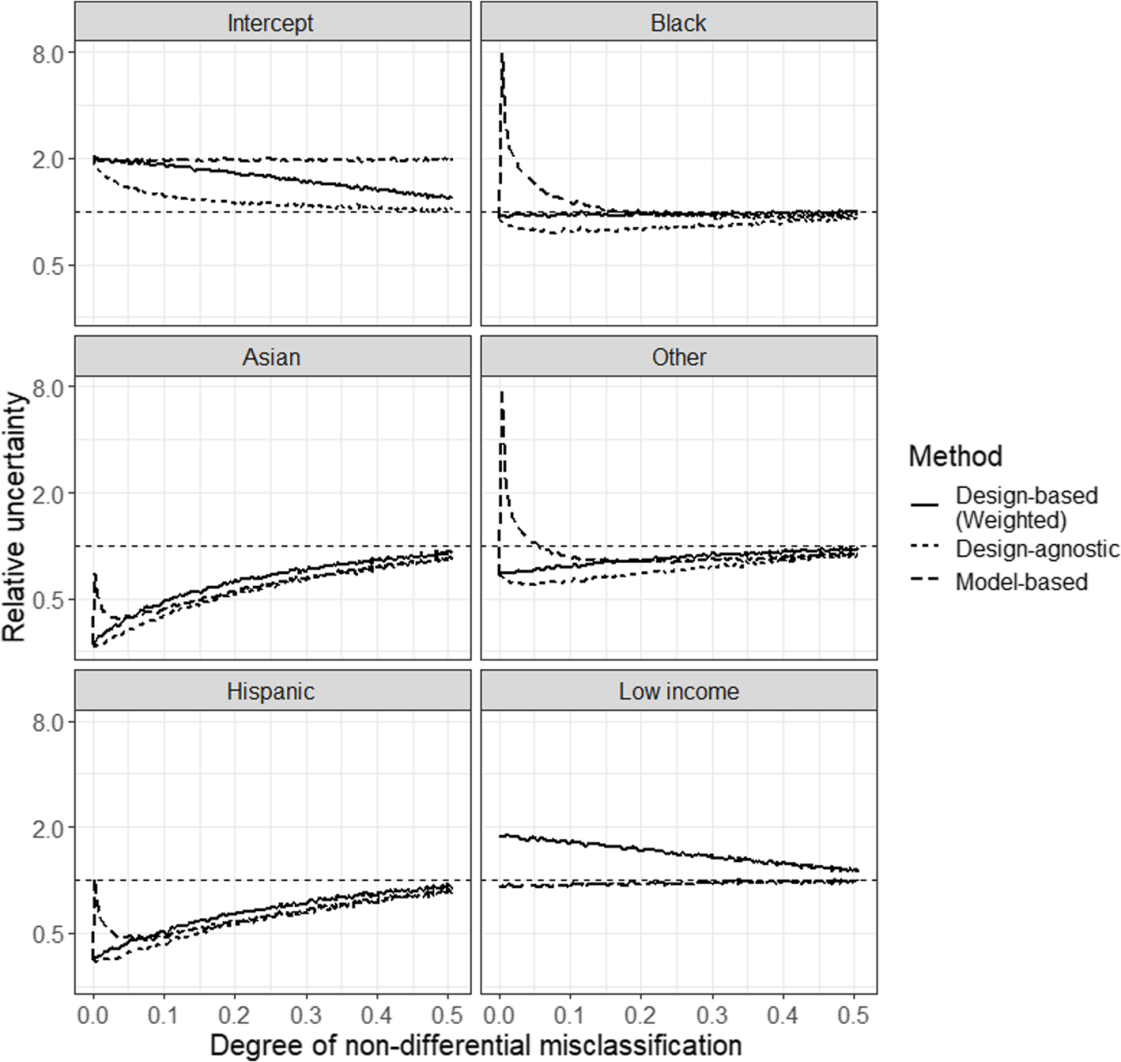
Relative uncertainty of design-agnostic, model-based and design-based methods under disproportionate stratified sampling compared to simple random sampling by degree of non-differential misclassification

Figure 2 shows the relative uncertainty (on a log2 scale) of each parameter estimates obtained from DSS at varying degrees of misclassification to those obtained from SRS. We present relative uncertainties of the design-agnostic (dotted line), model-based (dashed line) and design-based (solid line) methods. The relative uncertainty of the design-agnostic method is defined as $\frac {SE(\hat {\beta }_{\text {design-agnostic}})}{SE(\hat {\beta }_{\text {SRS}})}$ and similarly for the relative uncertainties of the model-based and design-based methods. Values less than one indicate that the design-agnostic, model-based or design-based method from DSS is more precise (smaller SE) compared to SRS. More specifically, if relative uncertainty is 0.5, then Wald-based confidence interval from the design-agnostic, model-based or design-based method from DSS is expected to be approximately half the width than that from SRS. The relative uncertainty by degree of misclassification for each variable in Eq. 4 is shown in each panel.

In the absence of misclassification, we observed considerably lower relative uncertainty in variables from less prevalent strata (Asian, Other, Hispanic) from DSS by all methods. In Black, Asian, Other and Hispanic estimates, relative uncertainties of the model-based method spike as the degree of misclassification increases from zero, then gradually decrease and eventually become similar to the relative uncertainties of the other two methods as misclassification further increases. This is because with very low degree of misclassification, the collinearity between *X* and *X*^∗^ is extremely high and a model that includes both *X* and *X*^∗^ as covariates, as in Eq. 2, will produce very large SEs. However, note that between the two unweighted methods (design-agnostic and model-based), relative uncertainty of the design-agnostic method is uniformly lower than that of the model-based method across various degrees of misclassification for each parameter estimate. We observed larger relative uncertainty in the intercept estimates with the model-based and design-based methods compared to SRS. With the design-agnostic method, we also observed greater uncertainty in the intercept estimate compared to SRS but the magnitude of relative uncertainty was less severe compared to the model-based or design-based method. These findings were not surprising because the intercept reflects the referent group (Whites) and under the DSS design, we sample fewer Whites. As for the non-sampling variable estimate, low-income, the uncertainties of design-agnostic and model-based methods were similar to that under SRS at each degree of misclassification. The uncertainty of the design-based method in the low-income estimate was higher compared to that under SRS at lower degrees of misclassification.

With the exception of relative uncertainty of the model-based method in the intercept, as the degree of misclassification increased from 0 to 0.5, the relative uncertainty of each variable converged to one. This is because the sample obtained from DSS starts to look more like the sample obtained from SRS as the degree of misclassification increases. In general, regardless of the analytical method, applying a DSS scheme resulted in less uncertainty (smaller SE) for associations with rare subgroups.

In summary, uncertainty in the point estimates from all three analytical methods were affected by the degree of non-differential misclassification. Generally, the uncertainty of less prevalent subgroups were smaller when employing DSS over SRS, especially with low degree of non-differential misclassification.

We make the following conclusions based on our simulation study. First, DSS can result in more precise sampling variable parameter estimates compared to SRS for the low prevalence subgroups. Second, validity of the parameter estimates and the size of SEs of the non-sampling variables are unaffected by the sampling scheme, modeling approach and the type of misclassification. Third, accounting for the sampling design by including the design-variable as a covariate (model-based method) did not produce better results compared to the design-agnostic method under any scenarios studied here. Finally, under non-differential misclassification, the design-agnostic method was preferred over the design-based method. Both methods yielded estimates with low biases but SEs were smaller for the design-agnostic method. However, under differential misclassification, the design-based method was preferred over the design-agnostic method (whose estimates were highly biased).

We can determine if misclassification is non-differential or differential by producing the misclassification tables stratified by the outcome of interest, as done in Table 1. If misclassification is non-differential (i.e. percentages of misclassification are similar between the outcomes), then we recommend employing the design-agnostic analysis. If misclassification is differential (i.e. percentages of misclassification are not similar between the outcomes), then we recommend employing the design-based analysis.

### Example: CERC study

We analyzed a subset of the data from the eMERGE CERC survey to investigate the effects of sampling stratum misclassification on various analytical approaches in a real-world setting. The primary goal of this survey was to learn about the factors associated with an individual’s willingness to participate in biobank-derived research. The present analysis aimed to estimate the association between trust in healthcare system and various patient characteristics including race/ethnicity and low-income. For this paper, we explored the data from Vanderbilt University Medical Center (VUMC). Among the 329,672 adult patients identified from the VUMC EHR, the researchers sent out the survey to 4,500 patients and 604 patients responded with complete self-report data. In the original CERC survey, the researchers anticipated this low response rate from the result of the pilot survey and estimated the number of surveys to mail out in order to obtain enough sample [6]. We recognize the implications of the low response rate, especially if it is related to the outcome of interest. However, non-response presents a distinct set of challenges and will not be address here. We refer our readers to other materials on this matter [19–21].

In the original study, the sampling strata consisted of as many as 288 levels that were based on the cross-classifications of age (<35, ≥35), gender (female, male), race (White, Black, Asian, Native American/Alaska Native, Hawaiian/Pacific Islander, Other), ethnicity (Hispanic, Non-Hispanic), education (less than high school diploma, high school diploma to some college, at least a college degree) and rural living (yes, no) [22]. For this paper, we combined some of the categories of race and ethnicity to create a new 5-level race/ethnicity variable with categories White, Black, Asian, Other and Hispanic, which resulted in 120 sampling strata.

Table 3 summarizes the survey respondent sample (unweighted) from VUMC. The relatively balanced percentages across the race/ethnicity categories among the survey respondents indicate that the attempt to enrich racial/ethnic minorities was successful. The distribution of gender, age and rural living appear balanced among the respondents. Twenty-seven percent of the respondents were categorized as low-income based on self-reported income brackets.

**Table 3:** Demographics of the Vanderbilt University Medical Center CERC respondent sample. Percentages [counts] are provided for each characteristic

	Survey response of 604 respondents, % [count]
**Gender**	
Male	45 [274]
Female	55 [330]
**Age in years**	
<35	26 [160]
35+	74 [444]
**Race/ethnicity**	
White	39 [237]
Black	18 [110]
Asian	13 [81]
Other	11 [67]
Hispanic	18 [109]
**Education**	
Less than HS	9 [53]
HS to some college	38 [230]
At least BS	53 [321]
**Rural living**	
Suburban/Urban	54 [327]
Rural	46 [277]
**Income in USD ($)**	
<30,000	27 [166]
30,000 to 59,999	23 [138]
60,000 to 149,999	33 [200]
150,000+	17 [100]

Table 4 shows the results from design-agnostic, model-based and design-based logistic regression analyses in which trust in healthcare system was regressed on self-reported race/ethnicity, low-income, age group, gender, education and rural living. For the design-based analysis, we truncated the survey weights at the 90th percentile, as was done in the original study [22]. The parameter estimates from the analysis using truncated weights were similar to those from the analysis using the full range of weights with no truncation. However, the standard errors from the analysis using truncated weights were smaller. Sensitivity analyses with weights truncated at the 100th, 99th and 95th percentiles are shown in Table S1 of Supplementary Material. We present the odds ratio (OR) and 95% confidence intervals (95% CI) from each method.

**Table 4:** Results from design-agnostic, model-based and design-based logistic regression analyses in which trust in healthcare system was regressed on self-reported race/ethnicity, low income, age, gender, rural living and education

	Design-agnostic	Model-based	Design-based
Variable	OR (95% CI)	OR (95% CI)	OR (95% CI)
**Race/ethnicity**			
White	1.00	1.00	1.00
Black	1.11 (0.66, 1.86)	1.42 (0.60, 3.33)	0.71 (0.26, 1.92)
Asian	0.80 (0.46, 1.38)	1.69 (0.71, 4.02)	0.69 (0.33, 1.44)
Other	0.76 (0.43, 1.35)	1.19 (0.61, 2.31)	1.23 (0.37, 4.07)
Hispanic	0.67 (0.41, 1.09)	0.78 (0.42, 1.47)	0.24 (0.08, 0.76)
**Low income**			
No (Income ≥$30,000)	1.00	1.00	1.00
Yes (Income <$30,000)	1.25 (0.81, 1.95)	1.24 (0.79, 1.95)	1.46 (0.56, 3.83)
**Age in years**			
≤35	1.03 (0.69, 1.52)	1.54 (0.58, 4.10)	0.87 (0.39, 1.94)
>35	1.00	1.00	1.00
**Gender**			
Male	1.00	1.00	1.00
Female	0.69 (0.49, 0.99)	1.23 (0.34, 4.45)	1.03 (0.49, 2.16)
**Rural living**			
No (Suburban/Urban)	1.00	1.00	1.00
Yes (Rural)	0.83 (0.59, 1.18)	0.82 (0.57, 1.17)	0.63 (0.31, 1.27)
**Education**			
Less than HS	0.96 (0.48, 1.92)	0.90 (0.43, 1.86)	0.90 (0.13, 6.37)
HS to some college	0.95 (0.64, 1.39)	0.93 (0.62, 1.39)	1.30 (0.58, 2.89)
At least college graduate	1.00	1.00	1.00

Consistent with our simulation results, the ORs and 95% CIs of some sampling variables including race/ethnicity, age and gender were quite different across the three methods. Also consistent with our simulation results, the ORs and 95% CIs of the non-sampling variable, low-income, were similar between design-agnostic [OR (95% CI) = 1.25 (0.81, 1.95)] and model-based analyses [OR (95% CI) = 1.24 (0.79, 1.95)]. The design-based analysis yielded OR (95% CI) = 1.46 (0.56, 3.83) for low-income, which is slightly higher compared to the other two methods although the difference is small in light of the estimates of uncertainty. Because we observed differential misclassification in our data, the design-based analysis is most viable. Compared to White patients, Black [OR (95% CI) = 0.71 (0.26, 1.92)], Asian [OR (95% CI) = 0.69 (0.33, 1.44)] and Hispanic [OR (95% CI) = 0.24 (0.08, 0.76)] patients were less likely to report trust in the healthcare system. The odds of reporting higher trust in the healthcare system were higher for patients with low income compared to those with higher income [OR (95% CI) = 1.46 (0.56, 3.83)].

## Discussion

In this paper, we characterized the precision gains from using a disproportionate stratified sampling scheme compared to using a simple random sample when the interest lies in making inferential statements regarding less prevalent subgroups, and the impact that sampling strata misclassification can have on the validity and relative uncertainty of various analytical methods for complex survey design.

Employing a disproportionate stratified sampling scheme was beneficial in producing more valid and precise parameter estimates of the less prevalent subgroups (i.e. racial/ethnic minorities). Because non-White racial/ethnic groups consisted of less than 20% of the overall patient population in our study, employing SRS to select the survey sample of size 2500 would have resulted in extremely few Black, Asian, Other, and Hispanic individuals. Furthermore, even if SRS scheme consisted of seemingly enough individuals to make meaningful inference of the less prevalent subgroups, with misclassification, the same does not apply for the “true” race/ethnicity categories. After we obtain the survey responses, we may learn that the sample consists of even fewer individuals in the rare subgroups due to misclassification. By sampling the same number of individuals from each race/ethnicity category regardless of the prevalence, we were able to make meaningful inference on each of the race/ethnicity subgroup employing the appropriate analytical method, even in the presence of misclassification.

We later learned, from the survey responses, that sampling strata were misclassified. We assumed that the self-reported race/ethnicity was the more reliable measure compared to the EHR-based race/ethnicity and estimated the effect of self-reported race/ethnicity on trust in the healthcare system. That is, for the purpose of analysis, we used “gold standard” as opposed to mismeasured variable values.

When designing a complex survey with potential misclassification in the sampling variable, understanding the type of misclassification (non-differential or differential) is crucial in choosing the correct analytical method to produce valid estimates. Through our simulation study, we learned that the design-agnostic method produces valid and more precise estimates compared to the model-based or design-based method when the sampling strata misclassification is non-differential on the outcome of interest. However, when the misclassification is differential, then only the design-based method produces valid inferences.

We considered the self-reported race/ethnicity along with other demographic information provided in the survey response as the gold-standard. The reason for why some individuals’ race/ethnicity in the EHR differed from the self-reported one is unclear but possible explanations include coding error and misinterpretation of the patient’s race/ethnicity by the healthcare professional. It is also possible that the self-reported race/ethnicity is incorrect since we do not know who completed the survey. However, our objective of this paper was to show the implications of misclassified sampling frame when the sampling variable is also a main predictor regardless of the reasons for misclassification.

Jang et al. [23] also encountered the issue of sampling strata misclassification based on race/ethnicity under complex survey design. The National Survey of Recent College Graduates conducted a survey to collect various information on recent graduates from bachelor’s or master’s degree programs in the United States. They employed a two-stage sample design in which schools were sampled in the first stage and students were sampled using a stratified sampling scheme within the sampled schools in the second stage. The stratification variables in the second stage were provided by the school and included degree cohort, degree level, field of major, race/ethnicity, and gender of the students. In the survey, the respondents provided their own race/ethnicity and some discrepancy was observed between the school-provided and self-reported race/ethnicity. In the analysis to estimate the number of graduates in each domain specified by the stratification variables in the second stage, the authors found that the effective sample sizes for Asian domains were less than anticipated in the design. As a consequence, they overestimated the graduation rates among Asians.

We acknowledge several limitations in our paper. First, we did not investigate the potential impact of differential non-response. The CERC survey had a low response rate (16%) and whether or not the patient responded may had been associated with their feeling of trust in the healthcare system, the main outcome of interest, and potentially resulting in biased estimates by race/ethnicity. Second, aside from the design-agnostic method, we only considered two analytical methods of survey data. Other approaches include raking [24, p. 139] and incorporating propensity score methods when the goal is to estimate effects of a certain exposure or treatment from complex survey samples [25–27]. The two methods investigated in this paper (design-based and model-based analyses) are the most commonly used analytical methods for complex survey and we believe that focusing on these two methods was a reasonable starting point to explore the effect of strata misclassification under the disproportionate stratified sampling scheme.

## Conclusion

In this paper, we investigated the consequences of sampling strata misclassification on the analysis of complex survey study, especially if the interest lies in making meaning inference about less prevalent subgroups. We found that the preferred method of analysis depends on the type of misclassification. If the sampling variable is non-differentially misclassified on the outcome of interest, then the design-agnositic method is preferable. All three methods that we examined produce valid estimates. However, the design-agnostic method produces the most precise ones. On the other hand, if the sampling variable is differentially misclassified, then the design-based method is preferable. Only the design-based method produces valid estimates in this case. Therefore, we recommend the readers to examine the type of misclassification before choosing the method of analysis. If the type of misclassification is unclear, then we recommend using the design-based method in order to obtain unbiased estimates even at the cost of slight inefficiency.

## Supplementary Information

**Additional file 1** Supplementary material.

## Data Availability

Data and analysis code, as well as code for the simulation study are available from https://github.com/ayamitani/MisclassSurvey.

## Abbreviations

CERC: Consent education regulation and consultation working group
DSS: Disproportionate stratified sampling
EHR: Electronic health records
SRS: Simple random sampling
