# Supplementary material for: Survey design and analysis considerations when utilizing misclassified sampling strata
Source: BMC Med Res Methodol. 2021 Jul 11;21:145. doi: 10.1186/s12874-021-01332-8 (PMC8273975; doi:10.1186/s12874-021-01332-8)
Supplement: Supplementary file 1 — Additional file 1 Supplementary material. [file 12874_2021_1332_MOESM1_ESM.pdf]

Supplementary material for "Survey design and analysis considerations when utilizing a misclassified sampling stratum"

Aya A. Mitani, Nathaniel D Mercaldo, Sebastien Haneuse, Jonathan S. Schildcrout

Table S1: Results from design-based logistic regression analyses with inverse probability of survey weights truncated at 100th(no truncation), 99th and 95th percentiles in which trust in healthcare system was regressed on self-reported race/ethnicity, poverty, age, gender, rural living and education

Variable	100th<br>OR (95% CI)	99th<br>OR (95% CI)	95th<br>OR (95% CI)
**Race/ethnicity**			
White	1.00	1.00	1.00
Black	0.87 (0.28, 2.73)	0.87 (0.28, 2.71)	0.73 (0.26, 2.04)
Asian	0.67 (0.30, 1.47)	0.67 (0.30, 1.47)	0.69 (0.32, 1.48)
Other	1.69 (0.43, 6.62)	1.69 (0.43, 6.63)	1.53 (0.42, 5.65)
Hispanic	0.20 (0.05, 0.81)	0.20 (0.05, 0.82)	0.20 (0.05, 0.76)
**Poverty**			
No (Income ≥$30,000)	1.00	1.00	1.00
Yes (Income <$30,000)	1.04 (0.22, 4.95)	1.05 (0.22, 4.98)	1.55 (0.51, 4.69)
**Age in years**			
≤35	1.05 (0.36, 3.04)	1.04 (0.36, 2.99)	0.88 (0.36, 2.13)
>35	1.00	1.00	1.00
**Gender**			
Male	1.00	1.00	1.00
Female	1.22 (0.44, 3.42)	1.23 (0.44, 3.43)	1.09 (0.48, 2.47)
**Rural living**			
No (Suburban/Urban)	1.00	1.00	1.00
Yes (Rural)	0.55 (0.22, 1.37)	0.55 (0.22, 1.36)	0.57 (0.26, 1.25)
**Education**			
Less than HS	0.24 (0.02, 2.80)	0.24 (0.02, 2.80)	0.52 (0.06, 4.58)
HS to some college	1.26 (0.41, 3.90)	1.25 (0.40, 3.88)	1.28 (0.53, 3.09)
At least college graduate	1.00	1.00	1.00
